# Clinical benefits of immune checkpoint inhibitors for malignant peripheral nerve sheath tumors with *NF2* mutation: a case report

**DOI:** 10.3389/fimmu.2025.1596348

**Published:** 2025-05-21

**Authors:** Yale Jiang, Guo Zhao, Qiyu Tang, Shujun Xing, Shuhang Wang, Ning Li

**Affiliations:** Clinical Cancer Center, National Cancer Center/National Clinical Research Center for Cancer/Cancer Hospital, Chinese Academy of Medical Sciences and Peking Union Medical College, Beijing, China

**Keywords:** MPNSTs, immune checkpoint inhibitors, immunotherapy, case report, rare cancer

## Abstract

**Background:**

Malignant peripheral nerve sheath tumors (MPNSTs), which arise from peripheral nerves or cells associated with nerve sheaths, are uncommon and biologically aggressive sarcomas. Currently, immune checkpoint inhibitors (ICIs) have exhibited antitumor efficiency in various sarcomas. However, there have been few reports on the clinical features and treatment response of ICIs in MPNSTs.

**Case presentation:**

We report a 22-year-old woman with an *NF2* pathogenic mutation and typical manifestations of MPNST. Her symptoms improved after the administration of intravenous anti-PD-1 monoclonal antibody and it demonstrated a sustained clinical benefit for 19.7 months. A literature review of four cases was included to emphasize the efficiency of ICIs in the treatment of MPNSTs. Four cases reporting ICI treatment in MPNSTs were identified using Web of Science. All the previous cases that received ICIs had reported a complete response regardless of PD-L1 expression and genetic predisposition, indicating potential efficacy in this rare and intractable tumor. Our case showed the sustained clinical benefit of anti-PD-1 monoclonal antibody in the uncommon tumor subtype harboring an *NF2* mutation as a first-line therapy after non-radical surgery despite the heavy tumor burden.

**Conclusion:**

Our case indicated that ICIs are warranted as first-line monotherapy in MPNSTs given the possibility of life quality improvement and durable clinical benefit.

## Introduction

Malignant peripheral nerve sheath tumors (MPNSTs) are aggressive soft tissue sarcomas that arise from the peripheral nerves or exhibit nerve sheath differentiation, accounting for approximately 5%–10% of all sarcoma cases (1). The morbidity rate of MPNSTs is less than 0.001%, which is equal in men and women, making it a rare tumor globally (2). Among individuals with neurofibromatosis type 1 (NF1) mutations, approximately 8%–13% develop MPNST, constituting nearly 50% of all MPNST cases. The remaining 45% of MPNSTs occur sporadically with unidentified genetic anomalies, while the rest may be attributed to radiotherapy (3). The median age of onset for patients with NF1-mutated MPNSTs is 26 years, with a median survival time of 1.3 years. Patients with disseminated MPNSTs have a median age of onset of 62 years and a median survival time of 2.8 years (4). Typical clinical manifestations of MPNSTs include palpable (or radiographically visible) masses involving the peripheral nerves, and loss of neurological function with or without pain (5, 6), which can result in loss of labor or disfigurement in young patients, severely affecting quality of life and imposing a heavy medical burden on society.

Thus far, complete surgery resection is the only curative means for MPNSTs. However, the postoperative local recurrence rate is high, approximately 40%–65%, and the distant metastasis rate is also considerable, approximately 40%–68% (7, 8). Moreover, there is no evidence-based support for the clinical benefit of neoadjuvant and adjuvant treatments, and whether to apply systemic chemotherapy and radiotherapy is controversial. Therefore, patients with MPNSTs have a poor prognosis, with 5- and 10-year survival rates of 34% and 23%, respectively, in the overall disease population. Chemotherapy for advanced metastatic MPNST has limited efficacy, with anthracycline-based combination chemotherapy, such as adriamycin and isocyclophosphamide, being recommended as the first-line therapy (9). However, findings from trials revealed that the median progression-free survival (PFS) of combined chemotherapy was only 4 months and the median overall survival (OS) was merely 13 months (9). A recent study reported a 5-year OS rate of 7.3% in patients with metastatic MPNSTs (10). Due to the poor response to chemotherapy and the fact that neither targeted therapy nor immunotherapy has gained conclusive efficacy in MPNSTs, the effective treatment of MPNSTs remains an unmet clinical problem that needs to be solved.

In this article, we present a patient with an MPNST who achieved durable benefit from an immune checkpoint inhibitor (ICI) and review relevant literature on the clinical characteristics and treatment responses of this condition.

## Case report

**Figure 1:**
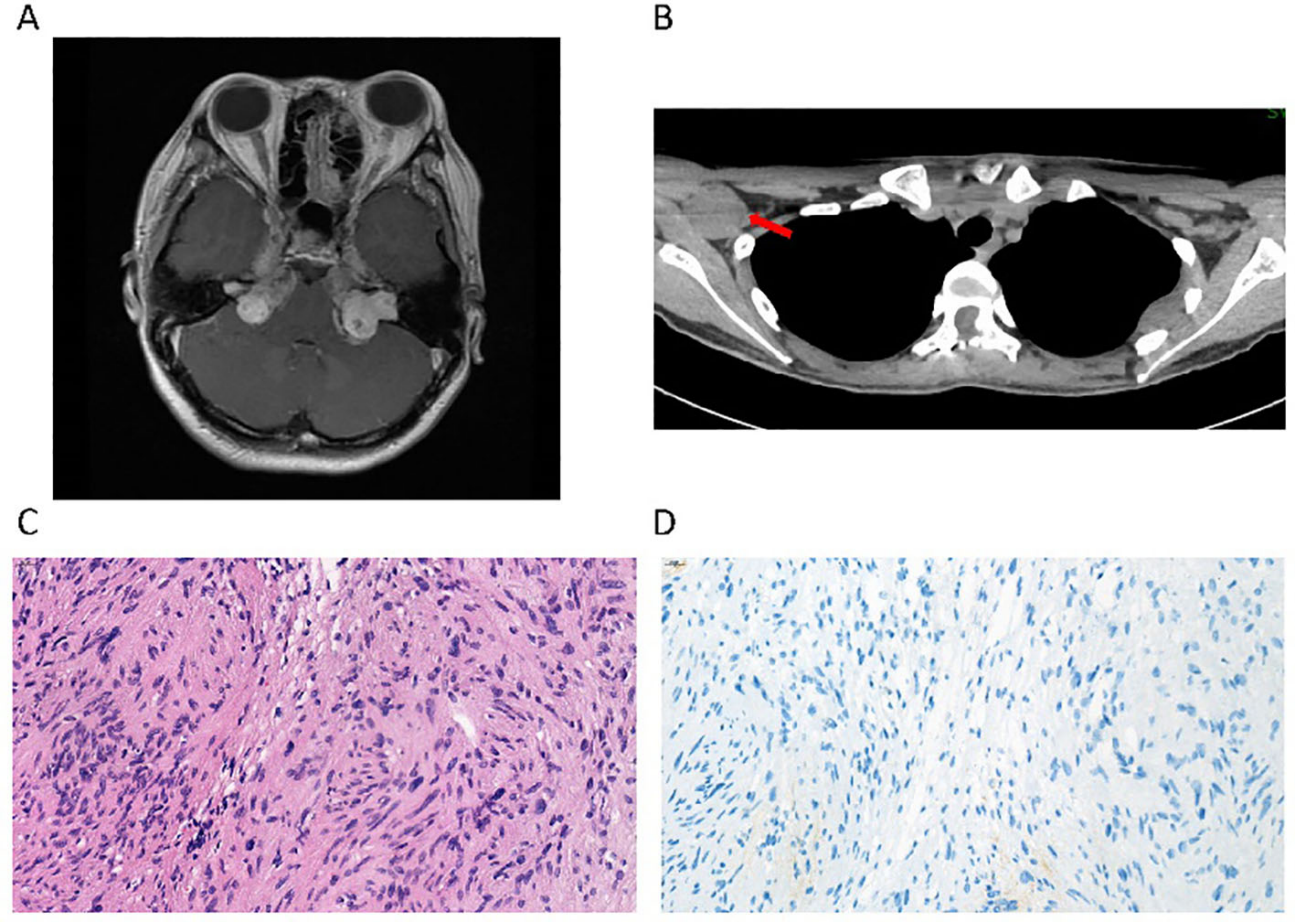
Radiological imaging and pathology of a patient with MPNST with an *NF2* mutation. **(A)** Brain magnetic resonance imaging (MRI) shows multiple masses with low signal on T1WI in the bilateral cerebellopontine angle regions, which is consistent with the typical manifestations of neurofibromatosis type 2. **(B)** Computed tomography (CT) shows a mass in the right axilla, which was observed to regress during the treatment with the anti-PD1 monoclonal antibody. **(C)** The pathological section of the resected spinal cord mass, stained with H&E, indicated cellular atypia. **(D)** The immunohistochemical staining of PD-L1 (22C3) was negative.

A 21-year-old woman presented in March 2022 with paresthesia in both lower limbs for 7 years. café-au-lait spots and soft masses were first observed when she was 2 years old. After that, she was misdiagnosed with subcutaneous lipoma for a long period and underwent two excision surgeries in her chest, neck, and head in her teenage years. After the second surgery, a dermatologist noted left eye proptosis and suggested ophthalmological examination. No apparent abnormality was found, and magnetic resonance imaging (MRI) of the nervous system was further recommended. Multiple abnormal signal masses and nodules were visible around the bilateral cerebellopontine angle regions, pons, and medulla oblongata (**Figure 1A**). Total resection via a craniotomy was performed considering her young age to avoid potential pressure on the optic nerves and irreversible loss of eyesight. The pathological examination indicated that she may have neurofibromatosis. Further whole-exome sequencing (WES) revealed a germline inactivating mutation at *NF2* c.536_545del (p.M179fs), which was pathogenic, but absent in both her parents. Combined with her clinical symptoms, a diagnosis of MPNST with neurofibromatosis type 2 was finally made.

After receiving her first dose of the COVID-19 vaccine in 2022, the masses continued to progress over the spine, causing unbearable pain. She underwent a fourth surgery for T4/5 spinal cord neurofibroma at a local hospital. Unfortunately, the surgery did not cure the pain. The tumor was occupying and squeezing the entire cervical spinal cord and had formed a cavity in the spinal cord. Reluctantly, she accepted the fifth surgery in C2–7 for resection of the ventricular meningioma, while the tumors located at the ventral part of the C1 medulla oblongata and below the T1–2 level were left untreated. The postoperative pathological examination suggested that a diffuse infiltrative growth of tumor cells could be seen within the spinal cord parenchyma, with a pseudorosette structure formed around the blood vessels (**Figure 1C**). The density of tumor cells was relatively high. Mitotic figures were frequently observed, approximately 4 per 10 high-power fields, and necrosis was also visible. Immunohistochemistry results showed GFAP (+), Olig-2 (-), Vimentin (+), S100 (+), H3K27Me3 (+), Ki67 (hot spot 30%+), and PD-L1(22C3) tumor proportion score (TPS) <1% (**Figure 1D**).

Unfortunately, the C2 cervical motor cord was injured during surgery, and she had to undergo exercise rehabilitation.

Her personal history was unremarkable. Nobody was observed to develop similar symptoms in her family. Physical examination revealed hyporeflexia in the lower extremities. She was able to stand with support. Cranial MRI showed multiple nodules in the cervical muscular space, cervical skin and subcutaneous, right supraclavicular region, bilateral pontine cerebellar peduncle region, cerebral pontine bridge, and peri medulla oblongata, and multiple nodular thickening of the cervical nerve roots, which is consistent with type 2 neurofibromatosis. Larger ones extended along the facial auditory nerve to the inner auditory canal.

This patient rejected chemotherapy and subsequently participated in an investigator-initiated phase 2 platform clinical trial treating rare tumors in China guided by molecular features (NCT04423185) in March 2022 (2). WES showed no actionable genetic alterations that could fit approved targeted therapies, and thus she was enrolled in the immunotherapy group and received treatment with sintilimab, a PD-1 inhibitor monotherapy. Dramatically, after 9 cycles of medication, she regained the ability to walk more than 30 meters unaided, with gradual improvement in self-care. A whole-body computed tomography (CT) scan showed that measurable lesions were stable without progression, in which tumor regression was noted in the right axillary nodule (**Figure 1B**). Repeated WES was conducted in this study to confirm the *NF2* mutation and indicated the status of microsatellite stability and low tumor mutation burden (TMB). Despite these seemingly unfavorable features, the clinical benefit persisted for 19.7 months until completion of 22 cycles of treatment, when a CT scan suggested that the right axillary node was noticeably more enlarged.

## Discussion

Multiple immune checkpoint targets that exert a negative regulatory effect on T cell antitumor response have been identified, including programmed cell death-1/programmed cell death-ligand 1 (PD-1/PD-L1) and cytotoxic T lymphocyte-associated protein 4 (CTLA-4). It is commonly observed that MPNSTs exhibit upregulation of checkpoint ligands, such as PD-L1, which provides a compelling rationale for investigating the potential benefits of targeting PD-1 and/or PD-L1 function in MPNSTs (15). Furthermore, MPNSTs have been verified to have a relatively higher level of infiltrating cytotoxic T cells and lower regulatory T cells (16). While these findings suggest MPNSTs may have an immune phenotype that may be responsive to immune checkpoint inhibitors (ICIs), the presence of tumor heterogeneity poses a clinical challenge for the application of immunotherapy treatment approaches.

Furthermore, a comprehensive literature search was performed in the PubMed and EMBASE databases until December 2023, resulting in the identification of four case reports pertaining to the use of immune-checkpoint inhibitors against MPNSTs (**Table 1**). Despite the limited number of individual case studies available, it is noteworthy that all the patients achieved a complete response. Detailed clinical information of the patient’s information, tumor location, previous treatment, PD-L1 expression status, treatment plan, and outcome are listed in **Table 1**. Payandeh et al. presented the case of an adult male patient with an MPNST with PD-L1 highly expressed and a TPS of 90%, who experienced complete remission of a mesenteric mass after receiving six cycles of pembrolizumab in combination with daily procarbazine (13). Davis et al. reported a patient with an MPNST with relatively low PD-L1 (2+, 5% of cells stained), who experienced a complete metabolic response after four cycles of single-agent pembrolizumab therapy (11). Another patient with an MPNST with 70% 2+ PD-L1 expression status also experienced a complete response to single-agent pembrolizumab after six cycles of therapy (14). Özdemir et al. reported a complete and prolonged response to nivolumab with radiotherapy in a patient with an MPNST with CD274/PD-L1 amplification (12). Similar to our finding, a patient with an MPNST enrolled in the phase I trial of pembrolizumab experienced disease progression after experiencing stable disease as the best response (17). Although these case reports indicate promising potential for the application of ICIs in the treatment of MPNSTs, a comprehensive evaluation of their safety and efficiency may prove challenging due to the relative rarity of this disease.

**Table 1:** Four published case reports of malignant peripheral nerve sheath tumors treated with immune checkpoint inhibitors.

Author/ study	Age (y)/sex	Tumor location (primary and metastatic)	Previous treatment	PD-L1 expression	Immunotherapeutic intervention	Treatment plan	Outcome
Davis et al. (11)	22/male	Osseous lesion of femoral head and neck with metastases in lung and pelvic lymph node	Surgical resection; radiotherapy	PD-L1 2 + 5% via IHC	Pembrolizumab	Intravenous; 21 cycles, 200 mg/3 weeks	Complete metabolic response
Özdemir et al. (12)	45/male	Peroneal nerve (left calf nodule) with lung and pleura metastases	Surgical resection; doxorubicin; ifosfamide	Almost 100% via IHC	Nivolumab (in combination with radiation therapy)	Nivolumab: 3 mg/kg/2 weeks; radiotherapy to metastases: 13 cycles at 3 Gy	Complete response
Payandeh et al. (13)	48/male	Retroperitoneal mass with mesenteric metastases	Surgical resection; doxorubicin and ifosfamide; imatinib; eribulin	Tumor proportion score: 90% via IHC	Pembrolizumab (in combination with procarbazine)	Pembrolizumab: 6 cycles, 200 mg/3 weeks/cycle; procarbazine hydrochloride: 50 mg/m2 twice a day	Complete remission
Larson et al. (14)	60/male	Paravertebral tumor at T7-T8 with lung metastases	Surgical resection; epirubicin and ifosfamide	70% (2+) via IHC	Pembrolizumab	Intravenous; 21-day cycle; 6 cycles; 200 mg for cycle 1–2; 400 mg for cycles 4–6	Complete response

IHC, immunohistochemistry; PD-L1, programmed cell death ligand protein 1.

The tumor microenvironment (TME) and immune landscape of MPNSTs have yet to be elucidated. Prior evidence suggested that PD-L1 expression was detected at a rate of 17% in the tumor tissue of MPNSTs, while 57% of the samples showed CD8+ T cell infiltration of no less than 1% in the TME (18). Another study, through bioinformatics analysis, revealed that the TME is composed of activated mast cells, cancer-associated fibroblasts, resting CD4+ memory T cells, and M2-like macrophages (19). These studies highlight that ICIs may be a potential candidate for patients with MPNSTs as an immune-based treatment. Furthermore, tumor immunogenicity could be enhanced through various combination strategies, including chemotherapy, targeted therapy, and oncolytic viruses, to stimulate tumor cell death and antigen release, boosting CD8^+^ T cell antitumor responses (20). Despite negative PD-L1 expression, the patient we reported carried a germline RAD51D mutation involved in the DNA damage response (DDR) pathway, which may serve as a predictive factor for positive immunotherapy outcomes (21).

The case report presented herein emphasizes the possibility of a sustained and measurable response to ICI treatment in individuals diagnosed with MPNSTs, thereby necessitating additional investigation in this disease. Notably, ongoing research endeavors include a phase II trial evaluating the efficacy of pembrolizumab in non-resectable patients with MPNSTs (NCT02691026), and two clinical trials exploring the potential of nivolumab in combination with ipilimumab in patients with rare tumors, including MPNSTs (NCT02834013 and NCT04465643). Furthermore, two phase I trials are ongoing, investigating MDM2-p53 inhibitor APG-115 in combination with pembrolizumab in patients with advanced solid tumors, including MPNSTs (NCT03611868), and nivolumab and BO-112, a nanoplexed form of polyinosinic:polycytidylic acid, before surgery for the treatment of resectable soft tissue sarcoma including MPNSTs (NCT04420975).

In conclusion, this case indicates that an ICI provided sustained clinical benefit as a first-line treatment for a patient with an MPNST with neurofibromatosis type 2. The application of immune monotherapy warrants more evidence from clinical trials.

## Data Availability

The original contributions presented in the study are included in the article/**Supplementary Material**. Further inquiries can be directed to the corresponding authors.
