# Supplementary material for: Clinical benefits of immune checkpoint inhibitors for malignant peripheral nerve sheath tumors with NF2 mutation: a case report
Source: Front Immunol. 2025 May 21;16:1596348. doi: 10.3389/fimmu.2025.1596348 (PMC12133985; doi:10.3389/fimmu.2025.1596348)
Supplement: Supplementary file 1 [file DataSheet1.pdf]

CARE Checklist of information to include when writin a case report

CARE case report guidelines

(cc) BY-NC-ND

Topic	Item	Checklist item description	Reported on Line
Title	1	The diagnosis or intervention of primary focus followed by the words “case report”	1-2
Key Words	2	2 to 5 key words that identify diagnoses or interventions in this case report, including "case report"	44
Abstract (no references)	3a	Introduction: What is unique about this case and what does it add to the scientific literature?	27-31
	3b	Main symptoms and/or important clinical findings	32-41
	3c	The main diagnoses, therapeutic interventions, and outcomes	32-41
	3d	Conclusion—What is the main “take-away” lesson(s) from this case?	42-43
Introduction	4	One or two paragraphs summarizing why this case is unique (**may include references**)	48-74
Patient Information	5a	De-identified patient specific information.	80-81
	5b	Primary concerns and symptoms of the patient.	82-87
	5c	Medical, family, and psycho-social history including relevant genetic information	110-116
	5d	Relevant past interventions with outcomes	94-100
Clinical Findings	6	Describe significant physical examination (PE) and important clinical findings.	130-132
Timeline	7	Historical and current information from this episode of care organized as a timeline	126-137
Diagnostic Assessment	8a	Diagnostic testing (such as PE, laboratory testing, imaging, surveys).	100-105
	8b	Diagnostic challenges (such as access to testing, financial, or cultural)	90-92
	8c	Diagnosis (including other diagnoses considered)	90-92
	8d	Prognosis (such as staging in oncology) where applicable	90-92
Therapeutic Intervention	9a	Types of therapeutic intervention (such as pharmacologic, surgical, preventive, self-care)	129-130
	9b	Administration of therapeutic intervention (such as dosage, strength, duration)	130
	9c	Changes in therapeutic intervention (with rationale)	126
Follow-up and Outcomes	10a	Clinician and patient-assessed outcomes (if available)	136-137
	10b	Important follow-up diagnostic and other test results	136-137
	10c	Intervention adherence and tolerability (How was this assessed?)	130-132
	10d	Adverse and unanticipated events	134-135
Discussion	11a	A scientific discussion of the strengths AND limitations associated with this case report	139-169
	11b	Discussion of the relevant medical literature **with references**.	139-169
	11c	The scientific rationale for any conclusions (including assessment of possible causes)	139-169
	11d	The primary “take-away” lessons of this case report (without references) in a one paragraph conclusion	139-169
Patient Perspective	12	The patient should share their perspective in one to two paragraphs on the treatment(s) they received	139-169
Informed Consent	13	Did the patient give informed consent? Please provide if requested	Yes ☑ No ☐
